# Mining cancer genomes in COSMIC

**DOI:** 10.1186/1753-6561-6-S6-P17

**Published:** 2012-10-01

**Authors:** Mingming Jia, Simon A Forbes, David Beare, Nidhi Bindal, Prasad Gunasekaran, Kenric Leung, Chai Yin Kok, Sally Bamford, Charlotte Cole, Sari Ward, Jon Teague, Michael R Stratton, Peter Campbell, Andrew Futreal

**Affiliations:** 1 Cancer Genome Project, Wellcome Trust Sanger Institute, Hinxton, Cambridgeshire CB10 1SA, UK

## 

COSMIC, The Catalogue Of Somatic Mutations In Cancer [http://cancer.sanger.ac.uk], is one of the largest repositories for somatic mutational events in human cancer. Data in COSMIC are curated from multiple sources, including from over 14,310 scientific publications, and are alongside data from the Cancer Genome Project at the Sanger Institute and global international consortia, such as The Cancer Genome Atlas and the International Cancer Genome Consortium. The COSMIC database currently accommodates over 300,000 mutations across 750,000 analyzed samples from 21,850 genes (COSMIC v60, July 2012). The Cancer Gene Census [http://cancer.sanger.ac.uk/cancergenome/projects/census/] is a list of almost 500 known cancer genes for which mutations have been identified as causally implicated in cancer. These genes are prioritized for full literature curation.

The collection of whole exome and genome sequencing data in COSMIC continues to grow at a rapid pace. There are: 17,614 coding mutations, 84,747 non-coding variants in 396 whole genome screens; 121,619 coding mutations and 12,949 non-coding variants as result of 1,266 full exome sequencing; 3,512 structural mutations derived from 77 rearrangement screens. The data overview for each whole genome screen is presented using Circos, for example, the NCI-H209 Circos summary [http://cancer.sanger.ac.uk/cosmic/sample/overview?id=688013].

Analyzing information from whole genome sequencing can greatly enhance the chance of discovering novel genes implicated in human cancer. Unlike hot spot screening of gene regions where somatic mutations are most frequent, the use of whole genome data can identify all mutations in all genes, providing much more expansive annotations to recurrence analysis as used to discover new cancer genes. For instance, there are recurrent somatic mutations identified in genes, for example: *SPOP* in 19 prostate samples; *SDK1* in 20 large-intestine samples.

There are several ways to access and analyze the data in COSMIC. The website allows data viewing in a genomic context supported by GBrowse while maintaining our gene-centric perspective. New additional features include a filter for excluding identified SNPs from the 1000 Genomes Project, and displaying Pfam domains and links to biological pathways for selected genes.

For mining a large dataset, COSMICmart (an instance of BioMart) is a tool for downloading user-customized datasets federated with external databases such as Ensembl and Uniprot. Moreover, we provide data export in multiple formats and Oracle database export through the FTP site [ftp://ftp.sanger.ac.uk/pub/CGP/cosmic].

In addition to somatic mutation data, we have integrated the data from the Genomics of Drug Sensitivity in Cancer Project [http://www.cancerrxgene.org], which is screening a wide range of anticancer therapeutics against over 1,000 genetically characterized human cancer cell lines.

Data analysis is becoming increasingly challenging due to the rapid expansion in cancer genome sequencing capacity. COSMIC is a major cancer genetics resource aiming to help such investigations, providing a centralized somatic mutations database with a wide suite of tools for its examination.

